# Correction: What makes a violent mind? The interplay of parental rearing, dark triad personality traits and propensity for violence in a sample of German adolescents

**DOI:** 10.1371/journal.pone.0280898

**Published:** 2023-01-20

**Authors:** The PLOS ONE Staff

## Notice of republication

This article was republished on December 27, 2022, to correct for errors in the Data Availability statement. The publisher apologizes for the errors. Please download this article again to view the correct version. The originally published, uncorrected article and the republished, corrected article are provided here for reference.

## Supporting information

S1 File Originally published, uncorrected article. (PDF)

S2 File Republished, corrected article. (PDF)
