# Supplementary material for: What makes a violent mind? The interplay of parental rearing, dark triad personality traits and propensity for violence in a sample of German adolescents
Source: PLoS One. 2022 Jun 22;17(6):e0268992. doi: 10.1371/journal.pone.0268992 (PMC9216556; doi:10.1371/journal.pone.0268992)
Supplement: S1 Appendix — (DOCX) [file pone.0268992.s001.docx]

Supporting Information for

“What makes a violent mind? The interplay of parental rearing, dark triad personality traits and propensity for violence in a sample of German adolescents”

Alexander Yendell, Vera Clemens, Julia Schuler and Oliver Decker

Appendix S1

Table S1. Sample characteristics

| Gender |  |
| --- | --- |
| Male | 545 (39.9%) |
| Female | 515 (37.7%) |
| Unknown Gender | 306  (22.4%) |
| Mean age (SD)  Range | 14.89 (0.89)  11-18 (93,7% at the age of 14 to 16) |
| **Dark triad** |  |
| Narcissism | 3.67 (1.84) |
| Machiavellianism | 3.06 (1.82) |
| Psychopathy | 3.32 (1.68) |
| Dark triad Core | 3.35 (1.52) |
|  |  |
| **Parental Rearing Behaviour** |  |
| Rejection & punishment (father) | 1.17 (0.43) |
| Emotional warmth (father) | 2.55 (0.84) |
| Control & overprotection (father) | 1.73 (0.65) |
| Rejection & punishment (mother) | 1.19 (0.42) |
| Emotional warmth (mother) | 3.05 (0.80) |
| Control & overprotection (mother) | 2.05 (0.71) |
|  |  |
| Propensity for violence | 1.9 (0.55) |

Sample Characteristics are presented as number of subjects (%) for gender and mean (SD) for other characteristics

Table S2. Comparison of own sample and data from the Saxony State Statistical Office, Leipzig, school year 2017/2018

| Type of school | Proportion of students (%) | | Deviation of relative shares  (in %) |
| --- | --- | --- | --- |
|  | Official Data | Sample |  |
| Secondary School | 39,8 | 39,3 | -0,5 |
| German Gymnasium | 35,7 | 45,2 | 9,6 |
| Special school | 4,5 | 3,9 | -0,6 |
| pre-vocational training year | 19,5 | 9,4 | -10,1 |
| Waldorf school | 0,6 | 1,8 | 1,2 |
| Hospital school | - | 0,3 | - |
| **Σ** | 100 | 100 | - |

Of the total of 4904 pupils in the 9th grade and the pre-vocational training year in Leipzig, 1366 students participated, corresponding to 27.9% of the students in the corresponding year. Non-participation at student level was mainly due to the absence of the students (e.g. due to illness, school absenteeism), as well as lack of parental consent or unwillingness of the students to participate. Compared with data from the Saxony State Statistical Office, Office for Statistics and Elections Leipzig, school year 2017/2018, for secondary schools, special schools and other types of schools there is hardly any deviation from the population, grammar school pupils are overrepresented in the present study. Female vocational school students, on the other hand, were recorded less frequently in Leipzig in relation to their actual frequency

Table S3. Propensity for violence: list of items

| I am prepared to use physical force against strangers. |
| --- |
| I would never use physical violence myself. But I think it's good when there are people who keep order in this way. |
| Physical violence against others is a normal part of human behaviour in order to get one's way. |
| Unfortunately, you have to resort to violence because that's the only way to get attention. |
| I am quite prepared in certain situations to use physical violence to assert my interests. |
| I would never use violence myself. But it's good that there are people who let their fists speak when there's no other way. |

Answer categories: not true at all, not true, true, completely true

Table S4:

Correlations of measurement errors

|  |  |  |  | Estimate |
| --- | --- | --- | --- | --- |
| e5 |  | <--> | e6 | 0.282*** |
| e5 |  | <--> | e3 | 0.233*** |
| e6 |  | <--> | e3 | 0.143*** |
| e6 |  | <--> | e4 | 0.128*** |
| e5 |  | **<-->** | e4 | -0.192*** |
| e9 |  | <--> | e7 | 0.183*** |
| e9 |  | <--> | e8 | 0.273*** |

***p<0.001
