# Supplementary material for: Correction: What makes a violent mind? The interplay of parental rearing, dark triad personality traits and propensity for violence in a sample of German adolescents
Source: PLoS One. 2023 Jan 20;18(1):e0280898. doi: 10.1371/journal.pone.0280898 (PMC9858313; doi:10.1371/journal.pone.0280898)
Supplement: S2 File — (PDF) [file pone.0280898.s002.pdf]

PLOS ONE

RESEARCH ARTICLE

What makes a violent mind? The interplay of parental rearing, dark triad personality traits and propensity for violence in a sample of German adolescents

Alexander Yendell[1☯*], **Vera Clemens**[2☯], **Julia Schuler**[1], **Oliver Decker**[3]

1 Research Center Social Cohesion, Section Leipzig University, Leipzig, Germany, **2** Department for Child and Adolescent Psychiatry/Psychotherapy, University of Ulm, Ulm, Germany, **3** Else-Frenkel-Brunswik-Institute, Leipzig University, Leipzig, Germany

☯ These authors contributed equally to this work.
* alexander.yendell@uni-leipzig.de

Check for updates

OPEN ACCESS

Citation: Yendell A, Clemens V, Schuler J, Decker O (2022) What makes a violent mind? The interplay of parental rearing, dark triad personality traits and propensity for violence in a sample of German adolescents. PLoS ONE 17(6): e0268992. https://doi.org/10.1371/journal.pone.0268992

Editor: Peter Karl Jonason, University of Padova, ITALY

Received: April 20, 2021

Accepted: May 12, 2022

Published: June 22, 2022

Copyright: © 2022 Yendell et al. This is an open access article distributed under the terms of the Creative Commons Attribution License, which permits unrestricted use, distribution, and reproduction in any medium, provided the original author and source are credited.

Data Availability Statement: All relevant data are within the paper and its supporting information files.

Funding: The authors Alexander Yendell, Oliver Decker and Julia Schuler received a grant by the Federal Ministry of Families, Seniors, Women and Youth (grant number BP 1302) (homepage of the ministry: https://www.bmfsfj.de/bmfsfj/meta/en). The funders had no role in study design, data collection and analysis, decision to publish, or preparation of the manuscript.

Abstract

Machiavellianism, narcissism and psychopathy are socially aversive personality traits that are strongly linked to the propensity of violence. A central determinate of aggression and violence is parental rearing. Interestingly, while the origin of the development of Dark Triad is not yet entirely understood, next to genetic and environmental factors, literature points towards an influence of parenting styles to the development of dark traits. Therefore, in a sample of 1366 9th grade students (mean age 14.89,), we assessed the interplay between parental rearing, dark triad traits, observation of violence among peers and their propensity for violence. The sample has a good representativeness on school types. Results reveal a positive association between the experience of parental rejection by both parents and punishment as well as parental control and overprotection and Machiavellianism, narcissism and psychopathy. Parental emotional warmth was associated negatively with Machiavellianism and psychopathy while no significant association with narcissism was seen. In a path model, parental rearing, dark triad traits and observation of violence among peers significantly contributed to the propensity of violence. However, differences between the experienced parenting behaviour of mothers and fathers should be noted. Both rejection and overly harsh punishments by fathers and emotional warmth by mothers have no significant influence on the dark triad. It is interesting that the effects regarding maternal parenting behaviour are stronger overall than the effects regarding paternal parenting behaviour. These results underline the importance of parental rearing on the development of Machiavellianism, narcissism and psychopathy and suggest a significant role of parental rearing and the dark triad traits on propensity for violence in adolescents. Parenting trainings and family interventions may be a promising starting point to prevent antisocial behavior linked to the dark triad and to prevent violent behavior in future generations

Competing interests: The authors have declared that no competing interests exist.

Introduction

Machiavellianism, narcissism and psychopathy are socially aversive personality traits which are collectively referred to as the "dark triad" of antisocial behavior [1]. Machiavellianism describes manipulative and strategic behavior in order to receive personal gain that goes along with coldness [2], but also social skills and charm that enable the prosocial behavior if needed [3, 4]. Narcissism refers to grandiosity, entitlement, and superiority [5]. It includes enhancing the self while devaluing others in order to maintain the positive self-view. However, the underlying factors for this are low self-esteem and vulnerability–even though the grandiosity aspect is much more apparent [6]. Psychopathy is characterized by a lack of emotions, empathy and remorse, but furthermore high impulsivity, low anxiety and thrill-seeking [1].

The Dark Triad refers to these traits on a subclinical and therefore non-pathological level [1]. Machiavellianism, narcissism, and psychopathy were developed as distinct traits but intercorrelate significantly [1, 7]. All three personality traits are linked to less agreeableness and conscientiousness [8], manipulation and callousness [9], lower self-control [10] and more superficial relationships [11]–factors that may contribute to antisocial behavior. In adults, the expression of all dark traits is associated significantly with the occurrence of aggression and delinquency [7]. For children around the age of 12 years, the occurrence of callous-unemotional traits predicted conduct problems, aggression and delinquency one year later [12]. Cross-sectional surveys showed associations between the Dark Triad [13], narcissism alone [5, 14] and callous- emotionality [5] and aggression in non-clinical adolescent samples. Furthermore, in a clinical sample of 6 to 12 years old children, an association between callous-emotionality and narcissism and aggression was seen [15].

Different underlying mechanisms are discussed to cause this increased aggression. For narcissism, there are hints that aggression is strongly linked to the feeling of shame [16] and vulnerability as it occurs when egotism is threatened [17]. For psychopathy, the lack of empathy and remorse increases aggressive behavior. Furthermore, impulsivity is a known risk factor for aggression as less self-control increases reactivity to provocations and a disinhibition in violating social rules [5]. Machiavellianism in school-age is associated to a lack of sympathy toward victims and bullying [18]. Taken together, there is sound evidence for a role of dark triad traits in antisocial and aggressive behavior [19].

The origin of the development of Dark Triad is not yet fully understood. Although genetic factors seem to increase vulnerability for Dark traits, the role of environmental factors seem to be pivotal [20, 21]. Importantly, a twin study showed individual differences in the highest levels of moral development to have no genetic basis but to be entirely attributable to environmental factors [20]. Literature points towards parenting styles to the development of dark traits to be an important envionmental factor [22]. Particularly the relevance of parenting on narcissism has been highlighted by Brummelman and colleagues [23]. There are mainly two hypotheses on the influence of parenting on the development of narcissistic traits. While social learning theory suggests children to develop narcissistic traits due to parental overwhelming, resulting in the childs' confidence to be special and to deserve privileges, psychoanalytic theory claims a lack warmth and appreciation towards children leading to overevaluation of the self as compensation. There is evidence supporting social learning theory [23, 24], but a lack of warmth has also been shown to be linked to narcissism [25].

However, also literature exists pointing towards an association between parenting and the other dark traits. High maternal care and secure parental attachment pattern have been shown to be associated negatively with the occurrence of psychopathy, Machiavellianism and Entitlement/Exploitiveness in narcissism while high paternal care was associated also negatively with psychopathy and Machiavellianism, but positively with narcissism [26]. Even though these

different results for paternal and maternal parenting and narcissism seems surprising, this study underlines the relevance of parenting for the development of dark triad traits. Literature points towards an effect of parenting on antisocial [27]. Longitudinal studies such as a 12-years prospective study by Luyckx and colleagues revealed increased antisocial behavior for indulgent and uninvolved compared to authoritarian and authoritative parents [28]. Interestingly, some literature points towards an influence of callous-unemotional behaviors and psychopathic traits on the association between parenting and conduct problems [29] and antisocial behaviors [30, 31]. Chinchilla and Kosson showed an association for parental warmness and conduct disorders only if adolescents revealed no psychopathic traits [30]. Hipwell and colleagues found a stronger association between harsh punishment and low parental warmth and conduct problems in the case of low compared to high callous-unemotional behavior [29]. This suggests a significant role of the dark traits in the association of parenting experience and antisocial behavior.

Another theory strand which promises a high explanatory power are theories of learning and subcultures. One the most prominent theories is differential association theory [29]. The theory which was developed by Sutherland states that criminal behavior is learned [32]. A person is likely to become delinquent if he or she comes across attitudes that favor violations of the law, and if these norms are stronger compared to attitudes that negatively assess violations. According to this theory, the contact mainly to criminals in a corresponding milieu leads to criminal behavior by learning the criminal behavior as a model. Referring to Sutherland's theory of differential associations, Aker's theory of social learning [33] raises the question of how delinquent behavior is learned.

The theory assumes that the learning of criminal behaviors depends on whether there is a positive or a negative stimulus due to deviating behavior. Delinquent behavior can either be punished in a group or milieu or amplified. According to the theory the latter leads to delinquent behavior. Another relevant theory is the subculture theory according to Cohen [34]. The theory assumes that crime is a consequence of the association of young people into so-called subcultures, in which normative values and morals prevail. However, it is also conceivable that the experience and observation of violence in a social milieu is not exclusively directly related to the advocacy of violence, but, just like negative parental rearing, has a negative influence on personality development.

Present study

Taken together, there is evidence for the influence of social learning on the development of dark triad traits and aggressive and antisocial behavior. The role of parenting is pivotal for social learning processes. Dark triad traits are a significant risk factor for antisocial behavior. However, to the best of our knowledge, studies assessing the role of dark traits in the relationship between experienced parenting and antisocial behavior are lacking to date. In the here presented study, we aimed to assess the interplay of parenting, the dark traits and a very distinct expression of antisocial behavior: the propensity for violence. We assumed that in addition to personality the learning of deviant behavior through observation in a corresponding violent milieu plays a role in explaining the propensity of violence. We hypothesized 1) negative parenting behavior to be associated with the development of dark traits and 2) the dark traits to be associated with the propensity of violence in our sample of adolescents.

Methods

Sample

Sampling. The survey took place between September 2017 and May 2018 and was conducted as a paper-pen-survey during school lessons. All general-education schools in city and

private sponsorship in Leipzig, which run a 9th grade, were asked to take part. In addition, in order to cover the young people of the age group who left general education school without a formal degree, all municipal and private schools leading to a vocational preparation year were also included in the sampling. The parents of the interviewees had to sign their consent beforehand.

A total of 33 schools, which corresponds 38.4% of all Leipzig schools with a 9th grade class or a preparatory year for vocational training, took part in the present study. Among them were 12 secondary schools, 9 grammar schools, 4 special schools, 6 vocational schools as well as one Waldorf and one hospital school. Since in some cases individual classes in the respective schools were not able to take part in the survey, the number of classes surveyed was 84, which corresponds to 34.4% of all classes in question. 39.9% of all respondents were male and 37.7 per cent were female. 22.4% of the respondents did not answer the question on gender. Further information on the representativity of the sample is given in S1 and S2 Tables in S1 Appendix. The questionnaire included questions on socio-demographics, observation of violence, victim experience, physical and mental attacks, delinquent behavior, general and politically motivated propensity and use of violence, narcissism, Machiavellism, psychopathology, experienced parenting styles and (traumatic) educational experiences. Characteristics of the final sample are given in the S1 Appendix. The study was audited by the Ethics Committee of the Medical Department of the University of Leipzig (398/17-ek).

Measures

Parenting. Parenting was assessed with a 9 item version of the Questionnaire of Recalled Parental Rearing Behaviour (QRPRB) [35], a shortened German version of the Swedish questionnaire "Egna Minnen av Barndoms Uppfostran" [36]. The QRPRB assesses the remembered parenting behavior retrospectively. The question is not aimed at a specific period of time, but at the complete recollection of the respondents. It encompasses the three factors "Rejection & punishment", "Emotional warmth" and "Control & overprotection", assessed each with three items, perceived separately for both, mother and father. As answers, participants can choose on a Likert scale with the categories 1 (no, never), 2 (yes, sometimes), 3 (yes, often) and 4 (yes, always). For our data set, Cronbach's alpha for "Rejection & punishment" was $\alpha = .81$ (father) and $\alpha = .75$ (mother), for "Emotional warmth" $\alpha = .78$ (father) and $\alpha = .81$ (mother) for "Control & overprotection" $\alpha = .60$ (both father and mother).

Narcissism, Machiavellianism, and psychopathy were assessed with the German version of the "Dirty Dozen", 12-item measure of the Dark Triad [37]. Each dimension is assessed with four items. As answers, participants can choose on a Likert scale with the categories 1 (not correct at all), 2 (rather not correct), 3 (rather correct) and 4 (fully correct). The questionnaire was shown to have a good internal consistency ($\alpha = .83$) [37]. In our analysis, the German version of the questionnaire was used [38]. The internal consistency of the questionnaire in our sample was high ($\alpha = .88$).

Propensity of violence was assessed by 6 questions regarding the general propensity for the use of violence (see S3 Table in S1 Appendix). For each item, participants can choose on a Likert scale with the categories 1 (not correct at all), 2 (rather not correct), 3 (rather correct) and 4 (fully correct). The individual items of the questionnaire are presented in the S1 Table in S1 Appendix. For the propensity of violence score, the mean of the answers was calculated. In our sample, the internal consistency was satisfactory ($\alpha = 0.73$). Observed violence was assessed by the question how often participants observed fight among adolescents in their neighborhood in the past 12 months.

Results

Correlation of parental rearing, dark triad traits and propensity of violence

Parental rejection and punishment as well as parental control and overprotection were positively associated with the expression of machiavellianism, psychopathy and narcissism. Parental emotional warmth was conversely associated with Machiavellianism and psychopathy and not associated with narcissism.

Rejection and punishment as well as control through the father as well as the mother were positively associated with machiavellianism, psychopathy and narcissism. Emotional warmth through the father as well as the mother was negatively correlated with machiavellianism and psychopathology but there was no significant association with narcissism.

All dark triad traits were positively associated singularly as well as combined with the propensity for violence.

Parental rejection and punishment as well as parental control and overprotection by the mother as well as the father were positively associated with propensity for violence while parental emotional warmth was conversely associated with propensity for violence. Not surprising but still interesting is that there were correlations between the upbringing behaviour of both parents. Against this background a possible danger that both parents will confirm each other even in the case of unfavourable parenting behaviour, so that the child possibly suffers under both parents.(for details see Table 1).

Results of the structural equation modeling

In the path model (see Fig 1), we were interested in the influence of the Dark Triad and the observation of violence (here: beating among young people.) With regard to the Dark Triad, we assumed that the score of the Dark Triad was influenced by parenting behavior. Overprotection and control as well as excessive punishment should be positively related to the Dark Triad, and emotional warmth should have a negative impact on the Dark Triad. In addition, we assumed that the observation of violence has not only a direct influence on the propensity for violence but also an influence on the Dark Triad. In the model, the covariance between the measurement errors were also taken into account (see S4 Table in S1 Appendix).

Model fit is excellent in terms of the root mean square error of approximation (RMSEA) with a value of <0.001 (lower interval limit <0.001). The R^2 is 0.196.

The results confirm the theoretical assumptions. The influence of the dark triad on the propensity of violence is a relatively strong direct factor that explains the propensity of violence (beta = 0.331, $p<0.001$). The observation of violence has a beta of 0.247 ($p<0.001$) and has also a small effect on the dark triad (beta = 0.104, $p<0.001$). All three queried dimensions of parenting experience play a significant role but there are slight differences between the rearing of the father and the mother. An upbringing shaped by control by the mother turns out to be the strongest influence on the Dark Triad (0.158, $p<0.001$). At the same time the effect of control through the father is weaker (0.060, $p<0.001$). The second strongest factor is rejection/punishment through the mother (0.111, $p>0.001$), whereas punishment by the father is not even significant. A weak effect is that of emotional warmth but only regarding the father (0.072, $p<0.001$).

Discussion

To our knowledge, this is the first study to examine the association among parental rearing behavior, Machiavellianism, psychopathy and narcissism and affirmation of violence. Our results suggest a significant role of parental rearing and the dark triad traits on propensity for

Table 1. Correlation of remembered parenting, dark triad and propensity for violence.

	Parental rejection & punishment (father)	Parental control & overprotection (father)	Parental emotional warmth (father)	Parental rejection & punishment (mother)	Parental control & overprotection (mother)	Parental emotional warmth (mother)	Machiavellianism	Psychopathy	Narcissism	Dark triad Core	Observation of violence	Propensity for violence
Parental rejection & punishment (father)	1	.243***	-197***	.399**	.151***	-.039 (n.s.)	.123***	.135***	.081**	.129***	.233**	.168***
Parental control & overprotection (father)		1	.122***	.198***	.521***	.069***	.147***	.115***	.162***	.166***	.136***	.087***
Parental emotional warmth (father)			1	-.080***	-.003 (n.s.)	.448***	-106***	-141***	-.006 (n.s)	-.098***	-.029 (n.s.)	-.098***
Parental rejection & punishment (mother)				1	.283***	-283***	.186***	.200***	.107***	.193***	.165***	.186***
Parental control & overprotection (mother)					1	.107***	.164***	.190***	.188***	.211***	.088**	.077**
Parental emotional warmth (mother)						1	-.070*	-.174***	-.002 (n.s.)	-.096***	-.047 (n.s.)	-101***
Machiavellianism							1	0.643***	0.628***	0.893***	0.178***	0.364***
Psychopathy								1	0.481***	0.820***	0.125***	0.311***
Narcissism									1	0.838***	0.067*	0.272***
Dark triad Core										1	0.145***	0.366***
Propensity for violence												1

*p<0.05;

**p<0.01,

***p<0.01. Furthermore, our results show that higher experience of parental rejection and punishment as well as parental control and overprotection is associated with Machiavellianism, psychopathy and narcissism.

https://doi.org/10.1371/journal.pone.0268992.t001

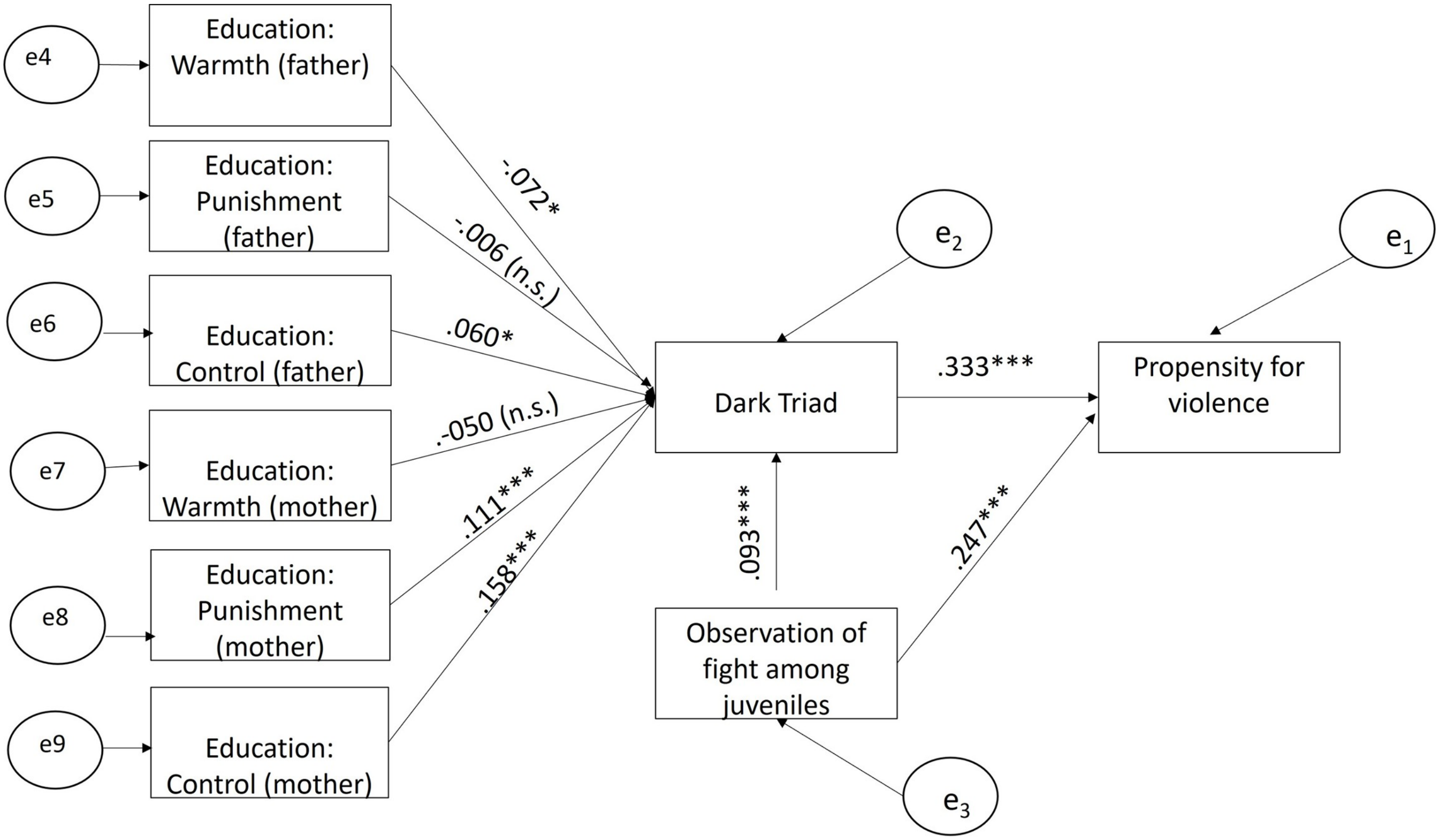

Fig 1. Parental rearing, dark triad personality traits and propensity for violence structural equation model.

https://doi.org/10.1371/journal.pone.0268992.g001

violence and thereby adds to the relevance of environmental factors, particularly experienced parenting style and social learning, in both, the development of antisocial traits and propensity of violence.

Parental rejection and punishment as well as parental control and overprotection by the mother and the father were associated with higher manifestation of Machiavellianism, psychopathy and narcissism while parental emotional warmth by both parents was associated conversely with Machiavellism and psychopathy but not significantly associated with narcissism.

As the family has significant impact on character development and primary social experience during childhood and consequently social learning, our result is not surprising. The relevance of the quality of parental care and parental attachment for the development of the dark triad traits were shown before [26]. Interestingly, in this study by Jonason and colleagues, high paternal care was associated also negatively with psychopathy and Machiavellianism, but positively with narcissism. In our analysis, parental emotional warmth and narcissism were the only two items that were not associated significantly with each other–underlining the potential complex role of parental emotional warmth for the development of the Dark Triad. There has been evidence before that narcissism is cultivated by parental overvaluation and not–as often stated in psychoanalytical theory–by a lack of parental warmth [22–24]. Therefore, our results seem to support rather social learning theory than the psychoanalytical concept. However, as we did not distinguish between parental warmth and overvaluation, this may explain the non-existing effect in our analysis.

Furthermore, our results show that higher experience of parental rejection and punishment by the mother as well as parental control and overprotection by both parents is associated with Machiavellianism, psychopathy and narcissism. Jia and colleagues argue unpredictable and harsh conditions to favor accelerated development and the focus on fast gain, which can go along with opportunistic actions, low empathy and social skills—common in individuals with high expression of dark traits [39].

Our results are in line with the results by Jonason and colleagues who show that lower quality of parental care and insecure or avoidant parental attachment patterns are associated with the Dark Triad traits [26]. As these results were assessed in adults, our study contributes that this association is already present in adolescence, pointing toward the relevance of parenting behavior before the age of 14 years for the development of the dark traits.

Consistent with previous research, antisocial Machiavellianism, psychopathy and narcissism were associated with propensity for violence [5, 12–15, 40]. In the next step, we analyzed the interplay between parental behavior, Dark Triad traits and propensity for violence. In line with a recent study in 530 Chinese students [41], our results point towards a significant role of the manifestation of the dark traits in the association between parental rearing and violence. Interestingly, the effects regarding maternal parenting behaviour are stronger overall than the effects regarding paternal parenting behaviour. An upbringing shaped by control by the mother turns out to be the strongest influence on the Dark Triad. At the same time the effect of control through the father is weaker. The second strongest factor is rejection/punishment through the mother, whereas punishment by the father is not significant. A weak effect is that of emotional warmth but only regarding the father. With the help of the study, we cannot conclusively answer why these differences in the experienced rearing of mothers and fathers exist. We can only speculate that this has to do with traditional family structures, and that on the one hand fathers draw boundaries and punish more often than mothers, which does not necessarily have a negative effect on character, and on the other hand mothers are more often perceived as warm anyway regardless of dark triad traits and perhaps in contrast with the father. Furthermore, we assume that the mother has a greater influence in parenting in families in the German context, not infrequently because many children of single parents in Germany on average spend more time with the mother than with the father.

The path model shows that 20% of the propensity for violence is explainable with parenting and dark triad traits, pointing towards the significance of this result. However, it is important to state that the observance of violence was shown to have a significant impact on the propensity for violence, too. As due to social learning, observation of violence is known to have a significant impact on the propensity for violence [29]. Nevertheless, our results point toward a significant interplay between parental rearing and the development of the dark traits for the affirmation of violence.

Limitations

First of all—as mentioned before—our study has a cross-sectional design. Therefore, no temporality can be assessed in the interplay between parental rearing, dark triad and propensity for violence. Reversal causality such as that manifestation of Machiavellianism, psychopathy and narcissism may impact the retrospective evaluation of parental rearing cannot be fully excluded, particularly as this was based on self-report and objective measures are missing. Moreover, only 20% of the propensity for violence was explainable by our model. Therefore, other factors such as character traits next to dark triad may play a significant role that were not assessed in our model. Even though the German version of the "Dirty Dozen" reveals good psychometric properties [37], the answers regarding dark traits

and the propensity of violence, may be influenced by social desirability and therefor bias the results. Character development is not finished at the age of 14 to 16. Thereby, our results are only limit generalizable for other groups, especially adults. Our sample included adolescents of different school types in order to achieve a good representativeness regarding socioeconomic and migration status. However, these characteristics were not questioned specifically. Due to many missings, gender was not considered in our model. In further studies the question should offer more than only the categories male/female. Another limitation concerns the scale for measuring parenting behaviour. It targets both the respondents' recollection and the subjective assessment of possible problematic behaviour of the parents. However, the intercorrelations between the different forms of parenting behaviour and the internal consistency point to a useful scale. However, further research methods are needed to obtain more precise research results. These could be extensive panel studies over a long period of time as well as evaluations from, for example, clinical studies with young people who are prone to violence. An unanswered question is why the parenting behaviour of mothers and fathers has a different impact on the Dark Triad. Our results point to a stronger influence of the mother. Whether this is because mothers may be more involved in parenting than fathers, at least in our survey at a German school, remains an open question. Another open question is what influence gender roles have on the development of the personality as well as on the relationship to violence. There is a need for further research here.

Nevertheless, our results give a significant contribution to the influence of parenting on the development of dark triad traits and the propensity for violence. In particular, they underline the role of harsh parenting and control. Therefore, parenting trainings and family interventions may be a promising starting point in order to prevent antisocial behavior linked to Machiavellianism, psychopathy and narcissism and most importantly, to prevent violent behavior in future generations. Moreover, the expression of Machiavellianism, psychopathy and narcissism are a distinct risk factors for propensity of violence that should be targeted in interventions for adolescents on risk for aggressive behavior. The discovered research gaps in our study allow for indications of possible fields of research. In addition to the early research on authoritarianism which focused on a coldly dominating father figure and traditional views of masculinity as a significant cause of (authoritarian) aggression. [42–45] future studies could also examine the impact on the absence of men or male role models outside the parental unit. Furthermore, in addition to abusive parenting styles, which are characterized by a lack of emotional warmth, verbal, physical and sexual abuse, parenting styles in which children are shown too few boundaries should be looked at. Such a parenting style could also have a negative influence on the development of the personality as well as make it difficult to deal with aggression and anger in a socially adequate and nonviolent way.

Supporting information

S1 Appendix.
(DOCX)

S1 Data.
(SAV)

Author Contributions

Conceptualization: Alexander Yendell, Julia Schuler, Oliver Decker.

Data curation: Alexander Yendell, Julia Schuler, Oliver Decker.

Formal analysis: Alexander Yendell, Vera Clemens.

Funding acquisition: Alexander Yendell, Oliver Decker.

Investigation: Alexander Yendell, Julia Schuler, Oliver Decker.

Methodology: Alexander Yendell, Vera Clemens, Oliver Decker.

Project administration: Alexander Yendell, Julia Schuler, Oliver Decker.

Resources: Oliver Decker.

Supervision: Alexander Yendell, Oliver Decker.

Validation: Alexander Yendell.

Writing – original draft: Alexander Yendell, Vera Clemens.

Writing – review & editing: Alexander Yendell, Vera Clemens, Julia Schuler.

References

1. Paulhus DL, Williams KM. The Dark Triad of personality: Narcissism, Machiavellianism, and psychopathy. Journal of Research in Personality. 2002; 36(6):556–63.
2. Christie R, Geis FL. Studies in Machiavellianism. New York: Academic Press; 1970.
3. Hawley PH. Prosocial and Coercive Configurations of Resource Control in Early Adolescence: A Case for the Well-Adapted Machiavellian 2003. 279–309 p.
4. Repacholi B, Slaughter V, Pritchard M, Gibbs V. Theory of mind, Machiavellianism, and social functioning in childhood. Individual differences in theory of mind: Psychology Press; 2004. p. 78–108.
5. Kerig PK, Stellwagen KK. Roles of Callous-Unemotional Traits, Narcissism, and Machiavellianism in Childhood Aggression. Journal of Psychopathology and Behavioral Assessment. 2010; 32(3):343–52.
6. Morf CC, Rhodewalt F. Expanding the Dynamic Self-Regulatory Processing Model of Narcissism: Research Directions for the Future. Psychological Inquiry. 2001; 12(4):243–51.
7. Muris P, Merckelbach H, Otgaar H, Meijer E. The Malevolent Side of Human Nature:A Meta-Analysis and Critical Review of the Literature on the Dark Triad (Narcissism, Machiavellianism, and Psychopathy). 2017; 12(2):183–204.
8. Veselka L, Schermer JA, Vernon PA. The Dark Triad and an expanded framework of personality. Personality and Individual Differences. 2012; 53(4):417–25.
9. Jones DN, Figueredo AJ. The Core of Darkness: Uncovering the Heart of the Dark Triad. European Journal of Personality. 2013; 27(6):521–31.
10. Jonason PK, Tost J. I just cannot control myself: The Dark Triad and self-control. Personality and Individual Differences. 2010; 49(6):611–5.
11. Jonason PK, Li NP, Buss DM. The costs and benefits of the Dark Triad: Implications for mate poaching and mate retention tactics. Personality and Individual Differences. 2010; 48(4):373–8.
12. Frick PJ, Cornell AH, Barry CT, Bodin SD, Dane HE. Callous-Unemotional Traits and Conduct Problems in the Prediction of Conduct Problem Severity, Aggression, and Self-Report of Delinquency. Journal of Abnormal Child Psychology. 2003; 31(4):457–70. https://doi.org/10.1023/a:1023899703866 PMID: 12831233
13. Muris P, Meesters C, Timmermans A. Some youths have a gloomy side: correlates of the dark triad personality traits in non-clinical adolescents. Child Psychiatry Hum Dev. 2013; 44(5):658–65. https://doi.org/10.1007/s10578-013-0359-9 PMID: 23334267
14. Fossati A, Borroni S, Eisenberg N, Maffei C. Relations of proactive and reactive dimensions of aggression to overt and covert narcissism in nonclinical adolescents. Aggress Behav. 2010; 36(1):21–7. https://doi.org/10.1002/ab.20332 PMID: 19918915
15. Fite PJ, Stoppelbein L, Greening L. Proactive and Reactive Aggression in a Child Psychiatric Inpatient Population:Relations to Psychopathic Characteristics. Criminal Justice and Behavior. 2009; 36(5):481–93.
16. Thomaes S, Bushman BJ, Stegge H, Olthof T. Trumping shame by blasts of noise: narcissism, self-esteem, shame, and aggression in young adolescents. Child Dev. 2008; 79(6):1792–801. https://doi.org/10.1111/j.1467-8624.2008.01226.x PMID: 19037950

17. Bushman BJ, Baumeister RF. Threatened egotism, narcissism, self-esteem, and direct and displaced aggression: does self-love or self-hate lead to violence? J Pers Soc Psychol. 1998; 75(1):219–29. https://doi.org/10.1037//0022-3514.75.1.219 PMID: 9686460

18. Sutton J, Keogh E. Social competition in school: relationships with bullying, Machiavellianism and personality. Br J Educ Psychol. 2000; 70 (Pt 3):443–56. https://doi.org/10.1348/000709900158227 PMID: 11059121

19. Moor L, Anderson JR. A systematic literature review of the relationship between dark personality traits and antisocial online behaviours. Personality and individual differences. 2019; 144:40–55.

20. Campbell J, Schermer JA, Villani VC, Nguyen B, Vickers L, Vernon PA. A Behavioral Genetic Study of the Dark Triad of Personality and Moral Development. Twin Research and Human Genetics. 2009; 12 (2):132–6. https://doi.org/10.1375/twin.12.2.132 PMID: 19335182

21. Vernon PA, Villani VC, Vickers LC, Harris JA. A behavioral genetic investigation of the Dark Triad and the Big 5. Personality and Individual Differences. 2008; 44(2):445–52.

22. Allroggen M, Ludolph A. Developmental psychopathology of narcissistic disorders. Psychotherapie, Psychosomatik, medizinische Psychologie. 2011; 61(11):453–8. https://doi.org/10.1055/s-0031-1291209 PMID: 22081463

23. Brummelman E, Thomaes S, Nelemans SA, Orobio de Castro B, Overbeek G, Bushman BJ. Origins of narcissism in children. Proceedings of the National Academy of Sciences. 2015; 112(12):3659–62. https://doi.org/10.1073/pnas.1420870112 PMID: 25775577

24. Cramer P. Young adult narcissism: A 20 year longitudinal study of the contribution of parenting styles, preschool precursors of narcissism, and denial. Journal of Research in Personality. 2011; 45:19–28.

25. van Schie CC, Jarman HL, Huxley E, Grenyer BFS. Narcissistic traits in young people: understanding the role of parenting and maltreatment. Borderline Personality Disorder and Emotion Dysregulation. 2020; 7:10. https://doi.org/10.1186/s40479-020-00125-7 PMID: 32426139

26. Jonason PK, Lyons M, Bethell E. The making of Darth Vader: Parent–child care and the Dark Triad. Personality and Individual Differences. 2014; 67:30–4.

27. Kochanska G, Barry RA, Aksan N, Boldt LJ. A developmental model of maternal and child contributions to disruptive conduct: the first six years. J Child Psychol Psychiatry. 2008; 49(11):1220–7. https://doi.org/10.1111/j.1469-7610.2008.01932.x PMID: 18684154

28. Luyckx K, Tildesley EA, Soenens B, Andrews JA, Hampson SE, Peterson M, et al. Parenting and trajectories of children's maladaptive behaviors: a 12-year prospective community study. J Clin Child Adolesc Psychol. 2011; 40(3):468–78. https://doi.org/10.1080/15374416.2011.563470 PMID: 21534057

29. Hipwell AE, Pardini DA, Loeber R, Sembower M, Keenan K, Stouthamer-Loeber M. Callous-unemotional behaviors in young girls: shared and unique effects relative to conduct problems. J Clin Child Adolesc Psychol. 2007; 36(3):293–304. https://doi.org/10.1080/15374410701444165 PMID: 17658975

30. Chinchilla MA, Kosson DS. Psychopathic Traits Moderate Relationships Between Parental Warmth and Adolescent Antisocial and Other High-Risk Behaviors. Criminal Justice and Behavior. 2016; 43 (6):722–38.

31. Edens JF, Skopp NA, Cahill MA. Psychopathic features moderate the relationship between harsh and inconsistent parental discipline and adolescent antisocial behavior. J Clin Child Adolesc Psychol. 2008; 37(2):472–6. https://doi.org/10.1080/15374410801955938 PMID: 18470783

32. Sutherland EH, Cressey DR. Principles of criminology. Philadelphia: Lippincott; 1966.

33. Akers RL. Deviant behavior: a social learning approach. Belmont, Calif.: Wadsworth Pub. Co.; 1985.

34. Bailey WC. Delinquent Boys: The Culture of the Gang. Albert K. Cohen. Glencoe, III.: The Free Press, 1955. 202 pp. $3.50. American Anthropologist. 1956; 58(1):215–6.

35. Petrowski K, Berth H, Schmidt S, Schumacher J, Hinz A, Brähler E. The assessment of recalled parental rearing behavior and its relationship to life satisfaction and interpersonal problems: a general population study. BMC Medical Research Methodology. 2009; 9(1):17. https://doi.org/10.1186/1471-2288-9-17 PMID: 19267894

36. Perris C, Jacobsson L, Linndström H, von Knorring L, Perris H. Development of a new inventory for assessing memories of parental rearing behaviour. 1980; 61(4):265–74.

37. Jonason PK, Webster GD. The dirty dozen: a concise measure of the dark triad. Psychol Assess. 2010; 22(2):420–32. https://doi.org/10.1037/a0019265 PMID: 20528068

38. Küfner A, Dufner M, Back M. Das Dreckige Dutzend und die Niederträchtigen Neun-Zwei Kurzskalen zur Erfassung von Narzissmus, Machiavellismus, und Psychopathie [The Dirty Dozen and the Naughty Nine—Short scales for the assessment of narcissism, Machiavellianism and psychopathy]. Diagnostica. 2015; 2015:76–91.

39. Jia X, Wang Q, Lin L. The Relationship Between Childhood Neglect and Malevolent Creativity: The Mediating Effect of the Dark Triad Personality. Front Psychol. 2020; 11(3566). https://doi.org/10.3389/fpsyg.2020.613695 PMID: 33391134

40. Hiemstra W, Verhulp EE, Thomaes S, Orobio de Castro B. Self-views and aggression in boys referred for disruptive behavior problems: self-esteem, narcissism, and their interaction. Eur Child Adolesc Psychiatry. 2019.

41. Liu G, Meng Y, Pan Y, Ma Y, Zhang D. Mediating Effect of Dark Triad Personality Traits on the Relationship Between Parental Emotional Warmth and Aggression. J Interpers Violence. 2019:886260519877950. https://doi.org/10.1177/0886260519877950 PMID: 31608785

42. Reich W. Die Massenpsychologie des Faschismus. Kopenhagen: Sexpol-Verlag; 1933.

43. Horkheimer M, editor. Studien über Autorität und Familie.: Forschungsberichte aus dem Institut für Sozialforschung. Paris: Librairie Félix Alcan; 1936.

44. Adorno TW, Frenkel-Brunswik E, Levinson DJ, Sanford RN. The Authoritarian Personality. New York: Harper und Brothers; 1950.

45. Theweleit K. Männerphantasien. Berlin: Matthes & Seitz Berlin; 2019. 1200 p. ger.
